# Clasp2 ensures mitotic fidelity and prevents differentiation of epidermal keratinocytes

**DOI:** 10.1242/jcs.194787

**Published:** 2017-02-15

**Authors:** Marta N. Shahbazi, Daniel Peña-Jimenez, Francesca Antonucci, Matthias Drosten, Mirna Perez-Moreno

**Affiliations:** 1 Epithelial Cell Biology Group, Cancer Cell Biology Programme, Spanish Cancer Research Centre (CNIO), Madrid 28029, Spain; 2 Experimental Oncology Group, Molecular Oncology Programme, Spanish Cancer Research Centre (CNIO), Madrid 28029, Spain

**Keywords:** Clasp2, Keratinocytes, Differentiation, Microtubules, Cell cycle

## Abstract

Epidermal homeostasis is tightly controlled by a balancing act of self-renewal or terminal differentiation of proliferating basal keratinocytes. An increase in DNA content as a consequence of a mitotic block is a recognized mechanism underlying keratinocyte differentiation, but the molecular mechanisms involved in this process are not yet fully understood. Using cultured primary keratinocytes, here we report that the expression of the mammalian microtubule and kinetochore-associated protein Clasp2 is intimately associated with the basal proliferative makeup of keratinocytes, and its deficiency leads to premature differentiation. Clasp2-deficient keratinocytes exhibit increased centrosomal numbers and numerous mitotic alterations, including multipolar spindles and chromosomal misalignments that overall result in mitotic stress and a high DNA content. Such mitotic block prompts premature keratinocyte differentiation in a p53-dependent manner in the absence of cell death. Our findings reveal a new role for Clasp2 in governing keratinocyte undifferentiated features and highlight the presence of surveillance mechanisms that prevent cell cycle entry in cells that have alterations in the DNA content.

## Abstract (precis)

**Highlighted Article:** Clasp2 is largely confined to the basal progenitor layer of the epidermis. This report shows that Clasp2 ensures mitotic fidelity and holds primary keratinocytes into an undifferentiated state.

## INTRODUCTION

Epidermal self-renewal is sustained by the presence of progenitor cells in the basal layer, which asymmetrically divide or delaminate, giving rise to the non-mitotic differentiated stratified layers (Blanpain and Fuchs, 2009; Lechler and Fuchs, 2005). Several molecular mechanisms are instrumental for the control of the finely tuned balance between proliferation and differentiation, including genetic and epigenetic changes, transcriptional regulation, signalling cues and cellular interactions (Blanpain and Fuchs, 2009; Simpson et al., 2011). Moreover, recent reports show that a mitotic block coupled to an increase in ploidy is associated with epidermal differentiation (Gandarillas and Freije, 2014; Zanet et al., 2010). Indeed, a ploidy increase is linked to cell differentiation during the development of multiple organs (Lee et al., 2009; Orr-Weaver, 2015), but despite its relevance, the molecular mechanisms involved remain poorly characterized.

Given the role of the microtubule (MT) cytoskeleton and some of its associated proteins during cell division and the reorganization of the microtubule network upon epidermal differentiation (Lechler and Fuchs, 2007; Sumigray et al., 2012), we have focused on the MT-binding protein Clasp2 as a candidate mediator of mitotic stress-induced epidermal differentiation. Mammalian Clasps (Clasp1 and Clasp2) are widely conserved MT plus-end-binding proteins that mediate MT stabilization (Akhmanova et al., 2001). In the context of mitosis, elegant reports have uncovered that CLASPs are fundamental for MT–kinetochore attachment, maintenance of spindle bipolarity, accurate chromosome segregation and spindle pole integrity, thereby preventing aneuploidy (Logarinho et al., 2012; Maia et al., 2012; Mimori-Kiyosue et al., 2006; Pereira et al., 2006). We have recently described that Clasp2 is largely confined to the basal progenitor layer of the epidermis, decorating the MT ends at cell adhesion sites (Shahbazi et al*.*, 2013; Shahbazi and Perez-Moreno, 2014). Here, we report that Clasp2 is not only essential to maintain epidermal architecture but also to ensure mitotic fidelity and maintain primary keratinocytes in an undifferentiated state.

## RESULTS AND DISCUSSION

### Loss of Clasp2 expression leads to premature differentiation of mouse and human basal keratinocytes

**Fig. 1.**
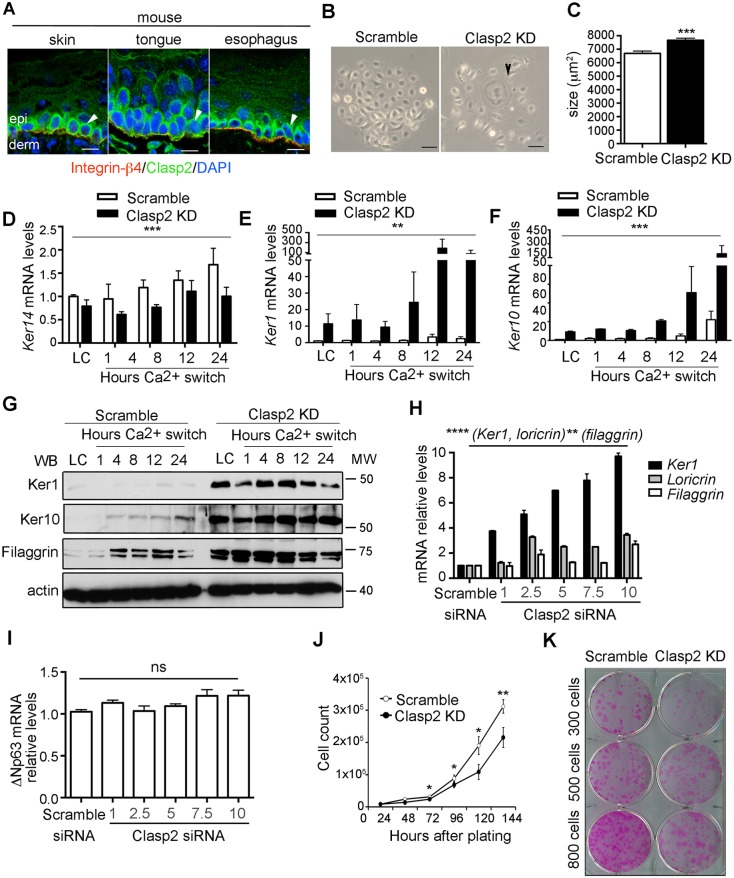
**Clasp2 expression in mouse keratinocytes prevents premature differentiation.** (A) Clasp2 localization in stratified epithelia (arrowhead). Epi, epidermis; derm, dermis. Scale bars: 10 μm. (B) Scramble and Clasp2KD mouse keratinocytes brightfield images. Arrowhead indicates a differentiated cell. Scale bars: 100 μm. (C) Quantification of cell size (*n*=562 scramble and 645 Clasp2KD cells). (D) *Ker14* mRNA levels in scramble and Clasp2KD mouse keratinocytes relative to levels of *Gapdh*. Hours Ca switch, time after Ca^2+^ switch. (E,F) *Ker1* and *Ker10* mRNA levels relative to that of *Gapdh* at different time points after Ca^2+^ addition. LC, low Ca^2+^. (G) Ker1, Ker10 and filaggrin immunoblots. (H) mRNA levels of differentiation genes relative to that of actin and (I) mRNA levels of ΔNp63 in scramble and mouse keratinocytes that had been treated with different concentrations (μM) of siRNAs against Clasp2 (Clasp2 siRNA). (J) Proliferation curves of scramble and Clasp2KD mouse keratinocytes. (K) Colony formation assay. Data are presented as mean±s.e.m. *P<0.02, **P<0.01, ***P<0.002 (C) Mann–Whitney U test, (D) two-way ANOVA test, (E,F) Kruskal–Wallis test, (H,I) one-way ANOVA test, (J) two-tailed Student's *t*-test; ns, non-significant. *n*=2–3 independent experiments per panel.

We have previously shown that Clasp2 is enriched in epidermal progenitor cells. This distribution differed from that observed for Clasp1, which appeared to be expressed across all epidermal layers (Fig. S1A). Interestingly, Clasp2 also localizes in the basal compartments of other mouse stratified tissues (Fig. 1A). This localization pattern was validated by performing peptide-competition assays and using alternative antibodies (Fig. S1B,C). Based on these findings, we hypothesized that Clasp2 is required to prevent the differentiation of epidermal keratinocytes.

To test our hypothesis, we used primary mouse and human keratinocytes as models, as they represent powerful *in vitro* systems that mimic the events of differentiation upon addition of Ca^2+^ to the medium (Hennings et al., 1980). We first knocked down Clasp2 in mouse keratinocytes using specific small hairpin (sh)RNAs. Immunoblot and real-time (RT)-PCR analyses confirmed the specific loss of expression of Clasp2 but not of Clasp1 (Fig. S1D,E).

Morphologically, control cells growing under proliferative low Ca^2+^ (LC) conditions exhibited a polygonal shape that was characteristic of undifferentiated mouse keratinocytes (Fig. 1B). In contrast, Clasp2 knockdown (Clasp2KD) cells displayed a squamous flat morphology and an increase in cell size (Fig. 1B,C); features that are associated with differentiation (Sun and Green, 1976). Immunoblot and RT-PCR analyses of the expression of keratins revealed that although Clasp2KD cells still expressed the basal markers *Ker14* (Fig. 1D) and ΔNp63 (an isoform encoded by *Tp63*) (Fig. 1I), high levels of the suprabasal postmitotic markers Ker1, Ker10 and filaggrin were expressed, even under LC conditions (Fig. 1E–G). To mimic the ∼50% reduction of *Clasp2* observed previously in the suprabasal epidermal layers *in vivo* (Shahbazi et al., 2013), we titrated different amounts of small interfering (si)RNAs specific for *Clasp2*. The expression of differentiation markers was readily apparent when the *Clasp2* mRNA levels were reduced to ∼30% (Fig. 1H; Fig. S1F), suggesting a causative role for Clasp2 in switching the mouse keratinocytes differentiation program. Interestingly, despite the conserved roles between Clasp1 and Clasp2, Clasp1 did not play an equivalent role in preserving mouse keratinocytes in an undifferentiated state (Fig. S1G,H).

The loss of Clasp2 was also accompanied by a significant decrease in cell proliferation (Fig. 1J) and clonogenic potential (Fig. 1K). We further validated our results in an immortalized mouse keratinocyte line, MCA3D (Navarro et al*.*, 1991). Clasp2KD MCA3D cells showed a flat and differentiated morphology (Fig. S2A) and an increase in the expression of differentiation markers (Fig. S2B,C).

**Fig. 2.**
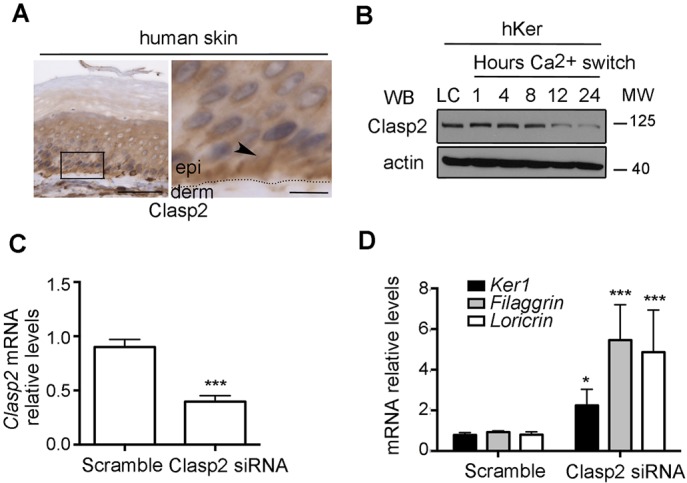
**Clasp2 expression in human keratinocytes prevents premature differentiation.** (A) Clasp2 localization in human skin (arrowhead). Scale bars: 50 μm (left); 10 μm (enlarged area on right). (B) Immunoblot of Clasp2 in human keratinocytes (hKer). LC, low Ca^2+^. (C) *Clasp2* mRNA levels in scramble and Clasp2 siRNA primary human keratinocytes. (D) mRNA levels of differentiation genes in scramble and Clasp2 siRNA primary human keratinocytes relative to levels of *Gadph*. Data are presented as mean±s.e.m. *P<0.02, ***P≤0.0006; ns, non-significant (Mann–Whitney U test); *n*=3–4 independent experiments per panel.

To determine whether Clasp2 plays a similar role in human keratinocytes, we first analyzed its localization in human skin samples. This revealed an enrichment of human Clasp2 in the basal progenitor layer (Fig. 2A). *In vitro* studies using primary human keratinocytes showed that Clasp2 levels decreased upon Ca^2+^ addition (Fig. 2B), indicating that, as in the mouse, Clasp2 expression is intimately coupled to the differentiation status of epidermal cells. Moreover, siRNA-mediated downregulation of *Clasp2* in primary human keratinocytes (Fig. 2C) led to an increased expression of differentiation markers (Fig. 2D). Interestingly, Clasp2 has been shown previously to be involved in hematopoietic stem cell maintenance (Drabek et al*.*, 2012), possibly through its role in regulating cell–matrix adhesions (Drabek et al*.*, 2012; Stehbens et al*.*, 2014). Although no alterations in focal adhesion proteins have been observed in Clasp2KD mouse keratinocytes (Fig. S2D–F), these results raise the possibility that Clasp2 sustains progenitor characteristics in different cellular contexts.

### Clasp2 expression ensures mitotic fidelity in primary mouse keratinocytes

**Fig. 3.**
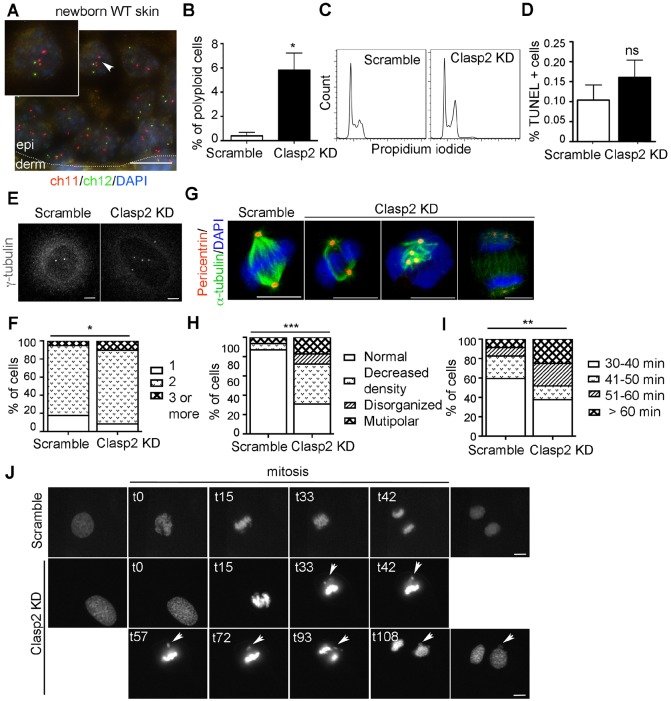
**Mitotic defects upon loss of Clasp2 in non-transformed mouse keratinocytes.** (A) FISH analysis for chromosomes (ch)11 and 12. Arrowhead indicates a suprabasal polyploid cell. Scale bar: 10 μm. (B) Percentage of polyploid mouse keratinocytes. (C) Scramble and Clasp2KD mouse keratinocytes cell cycle profiles. (D) Percentage of apoptotic cells (*n*=20 images per condition). (E) Staining of γ-tubulin in scramble and Clasp2KD mouse keratinocytes. Scale bars: 5 μm. (F) Quantification of centrosome number (*n*=193 scramble and 107 Clasp2KD cells). (G) Dividing scramble and Clasp2KD mouse keratinocytes were stained for pericentrin and α-tubulin. Scale bars: 10 μm. (H,I) Quantification of cells with the indicated spindle morphologies and the time spent in mitosis from cell rounding to completion of cytokinesis (*n*=68 scramble and 56 Clasp2KD cells). (J) Time-lapse images of H2B–GFP-expressing scramble and Clasp2KD mouse keratinocytes. The time after the initiation of DNA condesation is indicated. Arrowheads indicate lagging chromosomes. Scale bars: 10 μm. Data are presented as mean±s.e.m. (B,D). *P<0.04, **P<0.004, ***P=0.0001, ****P<0.0001; ns, non-significant; (B) two-tailed Student's *t*-test, (D) Mann–Whitney U test, (F, H, I) Fisher's exact test;. *n*=2 independent experiments per panel.

It has been recently shown that an increase in ploidy due to a mitotic block is associated with terminal differentiation in human epidermis (Gandarillas and Freije, 2014; Zanet et al., 2010). Using fluorescence *in situ* hybridization (FISH) assays, we confirmed the presence of some polyploid cells in the suprabasal layers of mouse skin (Fig. 3A), in agreement with previous observations (Karalova et al., 1988; Kartasova et al., 1992). In light of these findings and that a mitotic arrest (e.g. Taxol or Nocodazole treatment) is not sufficient to trigger differentiation (Fig. 3A), unless accompanied by an increase in DNA content (Freije et al., 2012), we hypothesized that the differentiation observed in Clasp2KD mouse keratinocytes stemmed from a mitotic defect leading to a DNA content increase. This is in line with the well-defined role of Clasp2 in the control of mitotic fidelity (Logarinho et al., 2012; Maia et al., 2012; Mimori-Kiyosue et al., 2006; Pereira et al., 2006).

To test this hypothesis, we first conducted cell cycle analyses and observed an increased proportion of polyploidy (Fig. 3B), as well as a high DNA content in Clasp2KD mouse keratinocytes (Fig. 3C; Table S1). This increase in the G2–M population was further validated using the sensors of the fluorescence ubiquitylation-based cell cycle indicator (FUCCI) (Fig. S3B): the monomeric Kusabira Orange (mKO2)–Ctd1 sensor of cells in G1 and the monomeric Azami Green (mAG)–Geminin sensor of cells in S, G2 or M phase (using the human proteins) (Sakaue-Sawano et al*.*, 2008). Importantly, this phenotype was not accompanied by an increase in apoptosis (Fig. 3D; Table S1).

We next analyzed whether the high DNA content observed in Clasp2KD mouse keratinocytes was associated with mitotic spindle alterations. Clasp2KD mouse keratinocytes exhibited a significant increase in centrosome numbers at interphase (Fig. 3E,F), and multiple mitotic spindle alterations, including decreased MT density, and multipolar and disorganized spindles (Fig. 3G,H). Time-lapse microscopy experiments showed that Clasp2KD mouse keratinocytes exhibited longer cell division times (Fig. 3I; Fig. S3C). These results were confirmed using mouse keratinocytes expressing histone-H2B–GFP (H2B–GFP). Several alterations were observed during mitosis, such as misaligned and lagging chromosomes (Fig. 3J), leading to inaccurate chromosome segregation.

### DNA damage and p53 activation are associated with the premature differentiation observed in Clasp2KD mouse keratinocytes

**Fig. 4.**
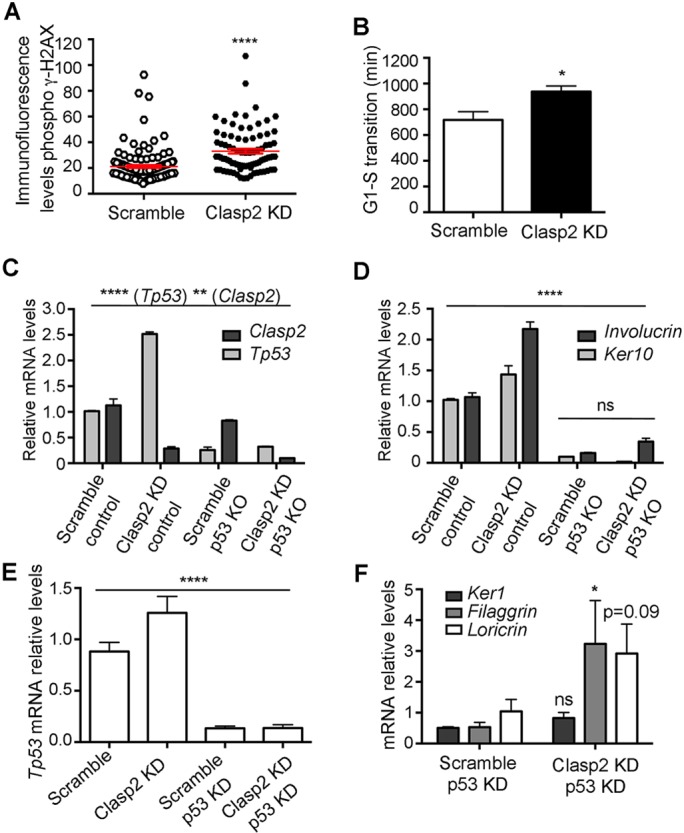
**DNA damage and p53 activation in the absence of Clasp2.** (A) Phospho-γH2AX immunofluorescence levels (*n*=124 scramble and 84 Clasp2KD cells) in mouse keratinocytes. (B) Time for which mouse keratinocytes coexpressed mKO2–Ctd1 and mAG–Geminin (*n*=30 scramble and 29 Clasp2KD cells). (C) mRNA levels of *Clasp2* and *Tp53* in mouse keratinocytes deficient for Clasp2 and p53. KO, knockout. (D) mRNA levels of the gene encoding involucrin and of *Ker10* in Clasp2- and p53-deficient mouse keratinocytes. (E) mRNA levels of *Tp53* in Clasp2- and p53-deficient human keratinocytes. (F) mRNA levels of differentiation genes in Clasp2- and p53-deficient human keratinocytes. Data are presented as mean±s.e.m. *P<0.05, **P<0.004, ****P<0.0001; ns, non-significant; (A,B) Mann-Whitney U test, (C) one-way ANOVA test (both for p53 and for Clasp2), (D) one-way ANOVA test, (E) Kruskal-Wallis test, (F) two-tailed Student's *t*-test. *n*=2 independent experiments per panel.

Alterations in chromosome numbers are known drivers of genomic instability and DNA damage (Passerini et al*.*, 2016). Accordingly, Clasp2KD mouse keratinocytes displayed significantly higher levels of DNA damage, as marked by the presence of phosphorylated (phospho)-γH2AX foci (Fig. 4A). Moreover, cell synchronization experiments revealed a delay in S-phase, in line with the observed increase in phospho-γH2AX and replication stress (Fig. S3D; Table S1). These results were validated by time-lapse microscopy studies of control and Clasp2KD mouse keratinocytes expressing the FUCCI sensors (Fig. 4B). Our results support a model in which Clasp2 deficiency induces mitotic alterations that instead of leading to cell death result in polyploidy and subsequent differentiation. Indeed, the induction of other mitotic alterations that result in polyploidy, such as inhibition of Aurora kinase A (AurkA) (Fig. S3E,F) or genotoxic agents that trigger a mitotic checkpoint (Freije et al., 2012), also lead to differentiation. Over time, the accumulation of chromosome alterations induces further genomic instability. Interestingly, loss of Clasp2, similar to the loss of AurkA (Katayama et al., 2004) or inhibition of other mitotic kinases (Freije et al., 2012) triggered an increase in p53 mRNA levels (*Tp53*; Fig. 4C) in response to DNA damage and mitotic checkpoint activation. A role for p53 in limiting the proliferation of polyploid cells has long been recognized (Ganem et al*.*, 2014), and in human keratinocytes its inactivation further potentiates squamous differentiation (Freije et al., 2014). To test if p53 has a similar role in the context of Clasp2 deficiency, we knocked down *Clasp2* expression in p53-null mouse keratinocytes (Fig. 4C) and in p53KD human keratinocytes (Fig. 4E). Clasp2KD p53KD human keratinocytes exhibited an increase in differentiation (Fig. 4F). However, Clasp2KD p53 knockout mouse keratinocytes showed a significant decrease in the expression of differentiation markers (Fig. 4D). These results underscore the existence of p53-dependent mechanisms in mouse keratinocytes that promote the differentiation of cells that bypass a mitotic alteration. However, loss of p53 in human keratinocytes triggers additional protective mechanisms that may not be conserved in mouse.

Our findings indicate that loss of Clasp2 in keratinocytes leads to reductions in cell growth due to mitotic alterations, leading to an increase in ploidy and premature differentiation in the absence of cell death, highlighting the presence of surveillance mechanisms in keratinocytes, which prevent the proliferation of cells with high DNA content and DNA damage. Although it is intriguing that the loss of Clasp2 does not cause apparent physiological defects in mouse skin (Drabek et al*.*, 2012), possibly due to compensatory mechanisms, overall our data indicate that Clasp2 is required to maintain the fidelity of cell division and to prevent the differentiation of mouse and human keratinocytes. Future research will shed light into how Clasp2 cooperates with cytoarchitectural, transcriptional and translational pathways to prevent keratinocyte differentiation.

## MATERIALS AND METHODS

### Primary cell culture, transfection, viral infection and treatments

Wild-type mice (C57/BL6) were handled according to the ethical regulations of the CNIO and the Institute of Health Carlos III, Madrid, Spain. Primary mouse keratinocytes were isolated from newborn mouse back-skin, as previously described (Shahbazi et al., 2013). MCA3D mouse keratinocytes (a gift from Amparo Cano, Biomedical Research Institute, Madrid, Spain) (Navarro et al., 1991) were cultured in Ham's F12 with 10% FBS.

p53 knockout mouse keratinocytes were generated by infecting mouse keratinocytes (Drosten et al*.*, 2014) with lentiCas9-Blast (gift from Feng Zhang, Massachusetts Institute of Technology, Cambridge, MA) and a pKLV-U6gRNA-PGKpuro2ABFP vector expressing p53sgRNA [gift from Sergio Ruiz, Spanish National Cancer Research Centre (CNIO, Madrid, Spain) (Ruiz et al., 2016) and maintained in CNT-07 (CELLnTEC, Bern, Switzerland).

Clasp2 expression was downregulated through lentiviral infection of a Clasp2-specific shRNA (Clone TRCN0000183632, Sigma) with 6 µg/ml polybrene, and stable clones were generated after selection with 400 µg/ml G418 (Calbiochem). Transient knockdown was achieved with four specific Clasp2 siRNAs (IDs SASI_Mm02_00299102-3, Sigma), and mouse keratinocytes were harvested after 72 h. Clasp1KD mouse keratinocytes were generated by transfecting an shRNA-pSuper plasmid against Clasp1 (gift from Anna Akhmanova, Utrecht University, The Netherlands) (Mimori-Kiyosue et al., 2005). For p53 knockout mouse keratinocytes and controls, cells were harvested 96 h after Clasp2 shRNA lentiviral infection.

For cell cycle arrest, mouse keratinocytes were treated with 30 µM nocodazole (M1404, Sigma) or with 1 µM taxol for 24 h. AurkA activity was inhibited with 10 µM MLN 8237 in DMSO (S1133, Selleckchem, Houston, TX).

Primary human keratinocytes (American Type Culture Collection, PCS-200-010) were cultured in CnT-57 (CELLnTEC, Bern, Switzerland). Clasp2 expression was downregulated using four specific siRNAs (IDs SASI_Hs01_00146296-97, Sigma) and Lipofectamine RNAiMAX (ThermoFisher). p53KD human keratinocytes were generated through lentiviral infection with a pLKO1shRNAp53 plasmid with 6 µg/ml polybrene.

Ca^2+^ switch experiments were conducted by switching cells from LC to normal Ca^2+^ (1.8 mM) medium. All cells were routinely tested for mycoplasma contamination.

### Cell cycle, proliferation and apoptosis analyses

Cell cycle analyses of live cells that had been stained with 10 mg/ml propidium iodide were conducted using a LSR FORTESA flow cytometer (Becton Dickinson) and the FlowJo software.

Cell cycle synchronization experiments, performed by blocking mouse keratinocytes at the G1/S boundary with a double thymidine block, and cell cycle profiles were analyzed at different time points after release from block.

To analyze cell cycle phases, mouse keratinocytes were infected with lentiviruses expressing the FUCCI sensors (Sakaue-Sawano et al*.*, 2008). For H2B–EGFP expression, mouse keratinocytes were transiently transfected with a KER14–H2B–EGFP vector (gift from Elaine Fuchs, The Rockefeller University, New York, NY) (Perez-Moreno et al., 2008).

For cell proliferation analysis, equal numbers of mouse keratinocytes were plated in triplicate. To analyze apoptosis, TUNEL-positive cells were detected using the In Situ Cell Death Detection Kit (Roche, Mannheim, Germany). For colony formation assays, cells were plated on fibronectin (Merck, New Jersey), fixed 1 week after plating and stained with Rhodamine B.

### RNA isolation and RT-PCR

Total RNA was isolated using TRIZOL (Invitrogen). cDNA synthesis was performed using Ready-to-Go You-Prime It First-Strand beads and random primers (GE Healthcare). RT-PCR reactions were performed using specific primers (Table S2), and expression levels were normalized to those of genes encoding actin or GAPDH.

### Immunofluorescence and immunohistochemistry

Optimal cutting temperature (OCT) compound-embedded frozen tissue sections or cells plated in coverslips were fixed in −20°C methanol for 3 min, blocked in blocking buffer (Shahbazi et al., 2013) and incubated with primary (Table S3) and secondary antibodies. Images were acquired in a Leica TCS-SP5 confocal microscope with the LAS-AF software.

For immunohistochemistry, formalin-fixed and paraffin-embedded skin sections were deparaffinized following standard protocols. For peptide competition assays, staining was performed in the presence of 10 μg GST (control) and 10 μg GST–Clasp2 (human; nucleotides 3074–3976 of the KIAA0627 cDNA as previously described) (Shahbazi et al*.*, 2013).

### Live-imaging microscopy

Mouse keratinocytes were plated onto 10 µg/ml fibronectin-coated glass-bottom culture dishes (Matek Corporation). Time-lapse experiments were performed in a Leica workstation AF6000 with controlled temperature and CO_2_ levels. Bright-field and FUCCI-expressing mouse keratinocyte images were captured every 5 min. Images of H2B–GFP-expressing mouse keratinocytes were captured every 3 min.

### Immunoblot

Cells were lysed in RIPA buffer and SDS-PAGE was performed using standard procedures.

### Fluorescence *in situ* hybridization

Probes RP23-324C12 and RP24-285E22 BACs (11qE1 band), and RP23-228E2 and RP24-386B9 BACs (2qH3 band) (Human BAC Clone Library, Children's Hospital Oakland Research Institute, Oakland, CA) were labelled by using a nick-translation assay with TexasRed and FITC, respectively. FISH was performed on paraffin tissue sections using the Histology FISH Accessory Kit (DAKO), denaturing samples at 66°C for 10 min, hybridizing probes at 45°C for 120 min, and washing samples with 20× saline-sodium citrate (SSC) buffer and 1% Tween-20 at 63°C before mounting.

### Quantification and statistical analysis

Image analyses were performed using ImageJ and Imaris software (Bitplane Scientific Software, Zurich, Switzerland). For statistical analysis of quantitative data, the data normality was evaluated with a Kolmogorov–Smirnov test. Data that presented a Gaussian distribution was analyzed using two-tailed Student's *t*-test or ANOVA. Otherwise Mann–Whitney and Kruskal–Wallis tests were used. Qualitative data were analyzed with a Chi-squared test. Statistical analyses were performed using GraphPad Software. All data are representative of at least two independent experiments performed in triplicate.
